# Mobile-Based Self-management Application Requirements for Patients With Gastric Cancer: Quantitative Descriptive Study of Specialist and Patient Perspectives

**DOI:** 10.2196/36788

**Published:** 2022-04-27

**Authors:** Azade Yazdanian, Hamed Mehdizadeh, Azita Balaghafari, Mahdi Kahouei, Maede Masoudinezhad

**Affiliations:** 1 Department of Health Information Technology School of Allied Medical Sciences Mazandaran University of Medical Sciences Sari Iran; 2 Department of Health Information Technology School of Allied Medical Sciences Semnan University of Medical Sciences Semnan Iran; 3 Technology Department Mazandaran University of Medical Sciences Sari Iran

**Keywords:** digital health, eHealth, telehealth, mHealth, mobile app, self-management, patient education, needs assessment, requirements analysis, stomach neoplasm, gastric cancer

## Abstract

**Background:**

Gastric cancer is one of the most common gastrointestinal cancers. Patients with gastric cancer experience disabilities and complications that lead to reduced quality of life. Empowering these patients by providing them with information and self-management skills can help reduce side effects and improve their quality of life.

**Objective:**

The aim of this study was to identify the user requirements for developing a mobile-based self-management app to support patients with gastric cancer.

**Methods:**

Data were analyzed using descriptive statistics and frequency distribution reports using IBM SPSS Statistics software.

**Results:**

All of the data elements and functional requirements except “data recording times” and “weight changes in graphs” were identified as essential by clinical experts and patients. Among the functional requirements required in a gastric cancer self-management app, the capabilities related to informing, announcing warnings, and reminders are included. In the demographic data section, most patients (14/26, 53%) did not comment on the importance of recording data such as name, surname, and place of residence, and the demographic data section was met with less agreement from patients than clinicians.

**Conclusions:**

Applying the requirements mentioned in this study can improve the self-management of patients with gastric cancer. Such apps can play an important role in empowering patients and improving their quality of life. However, the apps need to be designed and implemented to see how they can meet users’ requirements.

## Introduction

Cancer is one of the leading causes of death and disability worldwide, especially in developing countries. According to a report (GLOBOCAN), in 2020, 1 in 5 people were diagnosed with cancer during their lifetime, and 1 in 8 men and 1 in 11 women died due to the disease [1]. In some cases, cancer is caused not by a person's physical and genetic characteristics but by the person’s living environment [2-4]. Stomach cancer is now the fifth most common malignancy worldwide, after cancers of the lung, breast, colorectal, and prostate, with 1,033,701 new cases (5.7% of the total) estimated in 2018. It is the third most common cause of cancer-related death, with 782,685 deaths (8.2% of the total) in 2018 [5]. According to the National Cancer Institute, gastric cancer is very common in Japan, Central Latin America, South America, Eastern Europe, and parts of the Middle East [6]. In Iran, gastric cancer has been reported as the deadliest cancer. The statistics of disease incidence rates show the high incidence of gastrointestinal cancer in the northern provinces of the country, especially Mazandaran and Golestan [5,7].

Treatment for gastric cancer includes surgery, chemotherapy, and radiation therapy, which are used based on the stage of the disease. Surgery is a major and effective treatment in the early and advanced stages of the disease [8]. After gastrectomy surgery, there are many complications related to nutrition and gastrointestinal function. The most common of these complications are premature dumping syndrome, late dumping syndrome, and fat malabsorption, which can lead to gradual weight loss, premature satiety, abdominal pain, postprandial pain, and chronic diarrhea. Other nutritional problems that occur gradually include anemia, hypoxemia, vitamin C deficiency, and calorie and protein malnutrition, which have a significant impact on all aspects of the quality of life of these patients [9]. Self-management refers to the ability and autonomy of the individual to accept responsibility for self-care and to manage the physical, mental, and social consequences of having a chronic condition [10,11]. Today, self-management is performed by health care professionals through training booklets, audio and videotapes, and group meetings.

Currently, in the clinical environment, the most common types of patient education are using educational pamphlets, audiotapes, videos, and also oral presentations in personal or group sessions. These methods have low efficiency because they provide a large amount of information and rely on the individual's ability to recall information, which may lead to patient confusion. For example, about 40%-80% of oral information was immediately forgotten, and half of this information was not recalled correctly by patients [12]. Therefore, to solve these problems and limitations, new tools and approaches are needed, and smartphones are one of these suitable and well-known tools [13]. The advantages of using mobile health (mHealth) intervention include managing the improvement of the patient's condition during treatment and afterward, improving patient knowledge, self-management, drug management, and receiving social support from patients with similar conditions [14].

Therefore, due to the importance of self-management by patients with gastric cancer, the ineffectiveness of educational pamphlets and oral information, the role of smartphones in facilitating education and management [12,15,16], and the high prevalence of this cancer [1,5], the purpose of this study was to identify the requirements of mobile-based self-management app for patients with gastric cancer and help them to improve disease management.

## Methods

### Overview

This research was conducted using the quantitative descriptive method in 2021. The data collection tool in this study was a questionnaire designed by the research group (Multimedia Appendix 1). It was used to assess information needs and determine the data elements and capabilities required for a self-management app for patients with gastric cancer based on library studies, global guidelines for gastric cancer management and treatment, and searches of valid databases and scientific articles. This questionnaire was considered equal for the two groups of clinical staff and patients. The questionnaire consisted of 41 closed questions in 5 sections that included patient data (1 item), patient clinical data (8 items), disease management (6 items) and educational information (12 items), and app capabilities and functions in 3 areas of notices, program alerts, and reminders and screen capabilities of the program (14 items). At the end of the questionnaire, an open-ended question was asked to receive the participants' opinions on their issues. This questionnaire was designed based on a 5-point Likert scale. To determine the questionnaire content validity, the opinions of 5 experts in the field of cancer and information management were obtained, and the relevant corrections were made.

To determine questionnaire reliability, we used Cronbach alpha and invited 5 physicians and 10 patients with gastric cancer to participate. Cronbach alpha was 83% for the patient's individual data, 80% for the patient's clinical data section, 87% for the disease management section, 97% for the educational information section, and 92% for the app capabilities and functions section. Data analysis was performed based on the calculation of frequency distribution (number and percentage), mean, and quarter deviation index of each questionnaire question in IBM SPSS Statistics software (version 22; IBM Corp). Thus, if a total of 75% of the participants in the study or more chose the first two options (very important and important) in the questionnaire, that data element was considered in the final model, the data elements that a total of 50%-75% of the study population chose the first two options or the last two options in the questionnaire, were questioned again in the second stage of Delphi and comments below 50% of the model were removed. Thus, if a total of 75% of the participants in the study or more chose the first two options (very important and important) in the questionnaire, that data element was considered in the final model. The data elements for which 50%-75% of the study population chose the first two options or the last two options were included in the second stage of the Delphi process. Ultimately, comments below 50% of the model were removed. This study involved two rounds of the Delphi method. The scores of the questionnaire options were as follows: 5=very important, 4=important, 3=I have no opinion, 2=insignificant, and 1=very insignificant. In addition, if a new data element was suggested by at least 40% of the participants in the open question section of the questionnaire, the desired data element was used in the design of the program. Questionnaires were available to all medical specialists in the fields of cancer radiotherapy, blood and oncology, pathology, pharmacy, and head nurses of the chemotherapy department working in the hospitals of Mazandaran University of Medical Sciences, which had an oncology department (Bouali Sina and Imam Sari) and by available sampling to 30 patients with gastric cancer who were admitted to the oncology department and met the criteria for inclusion in the study. Inclusion criteria were technological skills, gastric cancer, smartphones, and a minimum age of 30 years and maximum age of 65 years. Finally, the obtained data were analyzed using descriptive statistics and frequency distribution reports and using IBM SPSS Statistics software.

### Ethical Considerations

This study was reviewed by Mazandaran University of Medical Sciences and provided with ethics code IR.MAZUMS.REC.1399.7855.

## Results

In the first round, 50 questionnaires (20 for clinical experts and 30 for patients) were distributed, and 42 questionnaires (16 by clinical experts and 26 by patients) were completed. In the second round, 50 questionnaires (20 for clinical experts and 30 for patients) were distributed, and 35 questionnaires (15 by clinical experts and 20 by patients) were completed. Most participants in the clinical group were male (n=10, 62%) and in the age range of 40-49 years (Table 1). Most of them had medical subspecialists degrees (n=9, 56%), and most of them (n=8, 50%) specialized in radiotherapy. In addition, most participants in this round (n=9, 56%) had work experience between 6 and 10 years. Most of the patient participants in the study were male (n=15, 57%) with a range of 50-59 years (Table 2). Most of them had a diploma (n=20, 77%), and most of them had a freelance job (n=8, 31%). Tables 1 and 2 show participants’ characteristics in the first and second rounds of the Delphi study.

**Table 1:** Clinical experts’ characteristics in the first and second rounds of the Delphi study.

Delphi study variables		Round 1, n (%)	Round 2, n (%)
**Gender**			
	Male	10 (62)	9 (60)
	Female	6 (38)	6 (40)
**Age (years)**			
	30-39	0 (0)	0 (0)
	40-49	12 (75)	11 (73)
	50-59	2 (12)	2 (13)
	60-69	2 (12)	2 (13)
**Level of education**			
	BSc	2 (12)	2 (13)
	MSc	0 (0)	0 (0)
	Medical specialty	5 (31)	5 (33)
	Medical subspecialty	9 (56)	8 (33)
**Occupation**			
	Oncologist	2 (12)	2 (13)
	Radiotherapist	8 (50)	7 (47)
	Pathologist	3 (19)	3 (20)
	Pharmacist	1 (6)	1 (7)
	Nurse	2 (12)	2 (13)
**Work experience in cancer (years)**			
	1-5	2 (12)	2 (13)
	6-10	9 (56)	8 (53)
	11-15	2 (12)	2 (13)
	≥16	3 (19)	3 (20)

**Table 2:** Patients characteristics in the first and second rounds of the Delphi study.

Delphi study variables		Round 1, frequency (%)	Round 2, frequency (%)
**Gender**			
	Male	15 (57)	12 (60)
	Female	11 (43)	8 (40)
**Age (years)**			
	30-39	0 (0)	0 (0)
	40-49	8 (31)	4 (20)
	50-59	10 (38)	10 (50)
	60-65	8 (31)	6 (30)
**Level of education**			
	Diploma	20 (77)	16 (80)
	Associate degree	3 (12)	1 (50)
	BSc	3 (12)	3 (15)
	MSc	0 (0)	0 (0)
	PhD or above	0 (0)	0 (0)
**Occupation**			
	Clerk	3 (12)	3 (12)
	Farmer	4 (15)	0 (0)
	Freelance job	8 (31)	12 (60)
	Retired	7 (27)	5 (25)
	Unemployed	4 (15)	0 (25)

Table 3 and Table 4 show participants’ response distribution regarding data elements and functional requirements. Most of the data elements were identified as very important or significant by the majority of participants (Table 3).

Among the data elements required in the patient's clinical data part, the highest mean was the treatment types used (mean 4.3, SD 0.91), and appointment time with doctor had the lowest mean (3.3, SD 1.66). Among the data elements required in the disease management part, the treatment protocols (ie, surgery, radiotherapy, and chemotherapy) had the highest mean (4.6, SD 0.95), and complementary therapies had the lowest mean (3.8, SD 1.17). Among the data elements required by the educational information, the highest mean belonged to physical activity (mean 4.4, SD 0.91), and the lowest mean belonged to excretory substances (mean 3.6, SD 1.18). Moreover, medication reminders had the highest mean (4.2, SD 0.95), and the nutrition reminders had the lowest average of the functional requirements for mobile-based self-management apps (mean 3.3, SD 0.48). However, some data elements led to the second round of Delphi, such as medication, other diseases and medications, appointment time with a doctor, excretory substances, list of cancer treatment centers, hopeful quotes notification, nutrition reminder, the ability to display the date entry time and date, and weight changes graph (Table 4).

In the second round, 15 specialists and 20 patients participated. Most of the participants in the study were male (n=9, 60%) and in the age range of 40-49 years (n=11, 73%). Most of them had a subspecialty degree (n=8, 53%), and most of them specialized in radiotherapy (n=7, 47%). In addition, most participants in this round (n=8, 53%) had work experience between 6 and 10 years. In addition, most of the patient participants in the study were male (n=12, 60%) and in the age range of 50-59 years (n=10, 50%). Most of them had a diploma (n=16, 80%), and most of them had a free job (n=12, 60%).

These results show that in the demographic data section, most patients did not comment on the importance of recording data such as name, surname, and place of residence and were met with less agreement from patients compared to clinicians. Furthermore, the group of clinical specialists emphasized the importance of recording the type of treatments, paraclinical measures (eg, laboratory tests, sonography, mammography, radiography, endoscopy, and cytology), and their results as well as introducing the side effects of chemotherapy and their medication interactions. Patients emphasized the importance of educational information such as nutrition management, emotional support, health advice during chemotherapy, and wound care after surgery. In terms of functional requirements, the patient group paid more attention to the necessary reminders for medication, visiting a doctor, and performing paraclinical procedures in the app, while experts emphasized the need for reminders to screen the patient's first-degree family. Moreover, the experts stated as the main treatment for gastric cancer is related to chemotherapy and surgery, so the list of surgeons and medical subspecialists in hematology and oncology should be considered in the field of informing capabilities of the app.

**Table 3:** Participants’ response distribution regarding required data elements for mobile-based self-management app (round 1).

Data elements		Patients, mean (SD)	Clinical specialists, mean (SD)	Total, mean (SD)	Agreement
Demographic data		3.7 (0.82)	4.2 (0.93)	3.8 (1.15)	✓
**Clinical patients data**					
	Occurrence of early symptoms (day/month/year)	3.92 (1)	4.4 (0.72)	4.1 (1.07)	✓
	Diagnosis time (day/month/year)	3.9 (0.88)	3.8 (1.14)	3.8 (1.02)	✓
	Paraclinical test history	3.9 (1.19)	4.36 (0.76)	4.1 (1.02)	✓
	Treatments type (surgery, chemotherapy, radiotherapy)	4.1 (0.98)	4.46 (0.74)	4.3 (0.91)	✓
	Medication	3.1 (1.57)	3.6 (1.66)	3.4 (1.59)	—^a^
	Other diseases and medications	3.1 (1.44)	3.7 (1.12)	3.4 (1.32)	—
	Appointment time with a doctor	3.1 (1.55)	3.6 (1.66)	3.3 (1.66)	—
	Time for paraclinical tests	3.85 (1.01)	4.6 (0.8)	4.2 (0.95)	✓
**Disease management**					
	Gastric cancer causes	4.0 (1.14)	4.5 (0.51)	4.2 (0.99)	✓
	Gastric cancer symptoms	4.1 (0.94)	4.7 (0.34)	4.3 (0.94)	✓
	Diagnostic methods (test, ultrasound, imaging, pathology)	3.7 (1.11)	4.1 (0.99)	3.9 (1.1)	✓
	Treatment protocols (surgery, radiation therapy, chemotherapy, etc)	4.2 (1.06)	4.8 (0.34)	4.6 (0.95)	✓
	Side effects and Medication interactions	4.1 (0.98)	4.4 (0.82)	4.3 (0.91)	✓
	Complementary therapies	3.7 (1.21)	4.0 (0.96)	3.8 (1.17)	✓
**Educational information**					
	Nutrition management	4.5 (0.51)	4.1 (0.96)	4.3 (0.8)	✓
	Risk factors	4.2 (1.04)	4.1 (1.32)	4.1 (1.10)	✓
	Excretory substances	3.4 (1.09)	3.8 (1.36)	3.6 (1.18)	—
	Rest	4.1 (0.99)	3.7 (1.11)	3.9 (1.13)	✓
	Stress management	4.2 (0.98)	4.1 (1.2)	4.1 (1.1)	✓
	Emotional support for patient and family	4.4 (0.82)	4.1 (1.02)	4.3 (0.91)	✓
	Physical activity management	4.6 (0.82)	4.3 (1.02)	4.4 (0.91)	✓
	Health advice during chemotherapy	4.1 (1.31)	3.7 (1.26)	3.9 (1.28)	✓
	Warning/danger symptoms during treatment (jaundice, bloody stools, bloody vomit)	4.1 (0.99)	3.7 (1.17)	3.8 (1.13)	✓
	Family education	4.0 (0.96)	3.9 (1.21)	3.9 (1.17)	✓
	Wound care after surgery	4.4 (0.82)	4.1 (0.98)	4.3 (0.91)	✓
	Frequently asked questions	3.9 (1.13)	4.4 (0.72)	4.0 (1.07)	✓

^a^No agreement reached.

**Table 4:** Distribution of the participants’ responses regarding functional requirements for mobile-based self-management app (round 1).

App functional requirements		Patients, mean (SD)	Clinical specialists, mean (SD)	Total, mean (SD)	Agreement
**Notices**					
	List of cancer treatment centers	3.6 (1.19)	3.3 (1.45)	3.5 (1.28)	—^a^
	List of cancer radiotherapists and hematologist-oncologist	4.3 (0.76)	3.9 (1.19)	4.1 (1.05)	✓
**Alerts and reminders**					
	Medication reminder	4.4 (0.81)	4.1 (1.09)	4.2 (0.95)	✓
	Appointment reminder	4.0 (0.92)	3.9 (1.13)	3.9 (1.03)	✓
	paraclinical test reminder	4.1 (0.99)	3.7 (1.17)	3.8 (1.09)	✓
	Screening reminder	3.6 (1.26)	4.1 (0.96)	3.9 (1.12)	✓
	Physical activity reminder	3.6 (1.26)	4.0 (0.92)	3.8 (1.15)	✓
	Hopeful quotes notification	3.6 (1.19)	3.2 (1.45)	3.4 (1.23)	—
	Nutrition reminder	3.0 (1.50)	3.6 (1.46)	3.3 (1.48)	—
**Display capabilities**					
	Ability to display data entry date	3.4 (1.36)	3.7 (1.39)	3.6 (1.38)	—
	Ability to display data recording time	3.3 (1.45)	3.7 (1.11)	3.5 (1.32)	—
	Show weight changes graphically	3.1 (1.50)	3.7 (0.98)	3.5 (1.23)	—
	Ability to record ultrasound images, test results, etc	3.9 (1.16)	3.8 (1.05)	3.8 (1.13)	✓
	Reports	4.1 (0.99)	3.7 (1.11)	3.9 (1.05)	✓

^a^No agreement reached.

## Discussion

### Principal Findings

The findings of this study showed that from the perspective of clinicians and patients, most components related to personal data, patient clinical data, disease management, and educational information, as well as app capabilities such as notices, alerts, and reminders, and screen-related capabilities other than “ability to display data recording hours and display weight changes in charts” were required. The findings of this study showed that most patients did not comment on the importance of recording data such as name, surname, and location, and personal data were met with less agreement from patients compared to clinicians. This may be due to concerns about privacy and confidentiality. Therefore, the results of this study are in line with the results of Neobek et al [17], who expressed users’ concerns about privacy as the main obstacle in using health-related self-management programs. In addition, the study of Malmi et al [18] also mentioned the importance of security and access to identity information in the design of apps. Therefore, due to the possibility of data transfer and communication with clinical specialists in apps, the existence of demographic information in self-management apps is essential.

In similar studies, the importance of recording patient clinical data was reported. In this regard, Sicotte et al [19] has shown that recording patient data and using electronic medical records led to improved flow of information, increased quality of care, and reduced the average waiting time in cancer outpatient centers. In addition, Yazdanian et al [20] stated it is possible to record patients’ clinical data electronically and manage the course of cancer from screening and prevention to treatment and beyond, despite the breadth of data elements related to cancer patient care. Levy et al [21] also created a form to collect data related to the chemotherapy protocol, assess pretreatment symptoms and provide chemotherapy training in the electronic health record. This form included data elements such as name, dose, and method of injection, as well as the expiration date of the medication. Mukai et al [22] also designed the Advanced Medical Information Database System (AMIDAS) to record clinical data and archive radiotherapy information. The data required by the AMIDAS system included patient demographic information, tumor data, radiotherapy treatment plan, follow-up (tumor complications, disease progression reactions, mortality, etc), laboratory results, and treatment delivery. Therefore, it seems that designing an app with a mobile phone will provide a complete view of patients with cancer by considering the types of data required by the oncology, chemotherapy, and radiotherapy departments, resulting in the integration of the data of patients with cancer, improving the quality of care, making more informed decisions, and reducing the time required to search for patient information.

The findings showed, due to the need for disease management, the presence of information on gastric cancer and its causes, symptoms, types of diagnostic methods, and treatment regimens are necessary. In a study conducted to develop a tablet-based app for patients with gastric cancer, Wu et al [15] found that patients had much less weight loss than the control group by providing sufficient information on the symptoms of the disease. In addition, Wu et al [23] designed a smartphone-based app that reminded activities related to nutritional status, medical information management, drainage follow-up, and wound care in patients with gastric cancer after surgery. This app informed patients of severe weight loss or possible bleeding by including clinical decision support. Ultimately, it achieved the highest level of satisfaction in 93% of users. Therefore, it seems that patients with gastric cancer need sufficient information about the causes and symptoms of the disease, diagnostic methods, and types of treatment regimens to improve their knowledge about the pathology and the course of the disease and the role and importance of treatment regimens.

Educational information was another major topic that many patients and clinicians emphasized, including the importance of nutrition management, stress management, health advice during chemotherapy, and more. In a similar study, June and Park [24] conducted a self-management program with 22 items in 7 areas of management of dietary restrictions, avoidance of risk factors, attention deposits, stress management and psychological support, attention to rest, regular diet, and follow-up care for patients with gastric cancer after gastrectomy. In this regard, Davoodi et al [9] emphasized the important role of the effect of self-care program training on the quality of life of patients with gastric cancer after surgery, especially in the psychological dimension. Moreover, Xuan [25] emphasized the very positive effect of self-management training on weight changes and quality of life in patients with gastric cancer that undergoing chemotherapy. In a review study, Mehdizadeh et al [12] found that mobile apps can provide easy access to appropriate and reliable information for patients with cancer and their families. Therefore, it seems providing educational information for supporting self-management by using mHealth intervention and mobile app can help patients with gastric cancer. It could be useful for nutrition management, diet therapy, improved physical activity, psychological and social effects, and sharing patients’ experiences with others.

Functional requirements related to informing, warnings, and reminders were functional requirements identified by participants as essential features for a gastric cancer self-management app. Some studies have reported that timely use of medications can lead to reduced disease recurrence and progression, reduced risk of mortality, and increased quality of life in patients with colorectal cancer. In this regard, Slatter et al [26] helped patients and their families by designing the ONCO FAMILY APP app, which had a reminder module for taking medication and seeing a doctor. In another study, Kock and colleagues [27] designed a LESS app with a calendar and reminder module for children with cancer. This module automatically reminds users of their appointments and periodic tests by specifying points on the calendar. Therefore, it seems mHealth interventions could be used as a promotional tool for encouraging people to participate in self-management activities and improving patient adherence to treatment protocols and communication between health care providers and patients.

### Limitations

This research had some limitations. First of all, although most medical specialists in the fields of cancer radiotherapy, blood and oncology, pathology, pharmacy, and head nurses of the chemotherapy department working in the teaching hospitals of Mazandaran University of Medical Sciences, which had an oncology department (Bouali Sina and Imam Sari), took part in the study, the number of the participants in the first and second rounds of the Delphi study was limited. However, as there is no well-defined rule for selecting a specific number of participants in a Delphi study and representation is assessed by the quality of the expert panel rather than its number, we can conclude that the participants were well-experienced clinicians in cancer care and the results might be generalized to larger sample sizes. The second issue might be related to the level of details associated with each data element. Although we reached a large number of data elements necessary for designing a mobile-based self-management app for patients with gastric cancer, it was not possible to include all of them in the questionnaire. Therefore, more details about other data elements, which might not be mentioned in this study, should be investigated before or during designing a real system.

### Conclusions

The goal of this study was to identify app requirements for the self-management of patients with gastric cancer. The features provided included personal data, patient clinical data, disease management, educational information, and functional requirements such as notifications and reminders that could be used for developing software or apps and made available for users. These apps can play an important role in empowering patients and also improving their quality of life. However, the apps need to be designed and implemented to see how they can meet users’ requirements.

## Appendix

Multimedia Appendix 1 Data elements and functional requirements questionnaire.

## Abbreviations

AMIDAS: Advanced Medical Information Database System
mHealth: mobile health
