# Supplementary material for: Mobile-Based Self-management Application Requirements for Patients With Gastric Cancer: Quantitative Descriptive Study of Specialist and Patient Perspectives
Source: JMIR Cancer. 2022 Apr 27;8(2):e36788. doi: 10.2196/36788 (PMC9096641; doi:10.2196/36788)
Supplement: Multimedia Appendix 1 [file cancer_v8i2e36788_app1.docx]

**Data Elements and Functional Requirements Questionnaire for Mobile-Based Self-Management Application for Gastric Cancer Patients**

**Part 1 : Demographic information**

Please fill this section:

1. Gender

2. Age

3. Level of education

4. Occupation

5. Work experience in cancer care

Part 2: Data elements and functional requirement

| Data elements | |  | | | | |
| --- | --- | --- | --- | --- | --- | --- |
|  |  | (1)  Very Insignificance | (2) Insignificance | (3)  I have no opinion | (4)  Important | (5)  Very important |
| Demographic data | |  |  |  |  |  |
| Clinical patients data | Occurrence of early symptoms (day / month / year) |  |  |  |  |  |
|  | Diagnosis time(day / month / year) |  |  |  |  |  |
|  | Para clinical test history |  |  |  |  |  |
|  | Treatments Type (surgery, chemotherapy, radiotherapy) |  |  |  |  |  |
|  | Medication |  |  |  |  |  |
|  | Other diseases and medications |  |  |  |  |  |
|  | Appointment time with doctor |  |  |  |  |  |
|  | Time for para clinical tests |  |  |  |  |  |
| Disease management | Gastric cancer causes |  |  |  |  |  |
|  | Gastric cancer symptoms |  |  |  |  |  |
|  | Diagnostic methods (test, ultrasound, imaging, pathology) |  |  |  |  |  |
|  | Treatment protocols (surgery, radiation therapy, chemotherapy, etc.) |  |  |  |  |  |
|  | Side effects and Medication interactions |  |  |  |  |  |
|  | Complementary therapies |  |  |  |  |  |
| Educational information | Nutrition management |  |  |  |  |  |
|  | Risk factors |  |  |  |  |  |
|  | Excretory substances |  |  |  |  |  |
|  | Rest |  |  |  |  |  |
|  | Stress management |  |  |  |  |  |
|  | Emotional support for patient and family |  |  |  |  |  |
|  | Physical activity management |  |  |  |  |  |
|  | Health advice during chemotherapy |  |  |  |  |  |
|  | Warning / Danger symptoms during treatment (jaundice, bloody stools, bloody vomit) |  |  |  |  |  |
|  | Family Education |  |  |  |  |  |
|  | Wound care after surgery |  |  |  |  |  |
|  | Frequently Asked Questions |  |  |  |  |  |

| Functional Requirements | |  | | | | |
| --- | --- | --- | --- | --- | --- | --- |
|  |  | (1)  Very Insignificance | (2) Insignificance | (3)  I have no opinion | (4)  Important | (5)  Very important |
| Notices | List of cancer treatment centers |  |  |  |  |  |
|  | List of cancer radiotherapists and hamate oncologists |  |  |  |  |  |
| Alerts and Reminders | Medication reminder |  |  |  |  |  |
|  | Appointment Reminder |  |  |  |  |  |
|  | Para Clinical test Reminder |  |  |  |  |  |
|  | Screening Reminder |  |  |  |  |  |
|  | Physical Activity Reminder |  |  |  |  |  |
|  | Hopeful quotes Notification |  |  |  |  |  |
|  | Nutrition reminder |  |  |  |  |  |
| Display capabilities | Ability to display data entry date |  |  |  |  |  |
|  | Ability to display data recording time |  |  |  |  |  |
|  | Show weight changes graphically |  |  |  |  |  |
|  | Ability to record ultrasound images, test results, etc. |  |  |  |  |  |
|  | Reports |  |  |  |  |  |
